# Clinical characteristics and prenatal diagnosis for 22 families in Henan Province of China with X-linked agammaglobulinemia (XLA) related to Bruton’s tyrosine kinase (BTK) gene mutations

**DOI:** 10.1186/s12881-020-01063-5

**Published:** 2020-06-17

**Authors:** Shanshan Gao, Shuang Hu, Huikun Duan, Li Wang, Xiangdong Kong

**Affiliations:** The Genetics and Prenatal Diagnosis Center of the First Affiliated Hospital of Zhengzhou University (Zhengzhou, China), No. 1, Jianshe East Rd, Erqi District, Zhengzhou, Henan Province China

**Keywords:** XLA, *BTK*, Gene mutations, Prenatal diagnosis

## Abstract

**Background:**

X-linked agammaglobulinaemia (XLA) is a rare immunodeficiency disease for which recurrent severe infection is the major clinical symptom. *BTK* is the main causative gene, with X chromosome recessive inheritance. However, the mutations reported to date do not fully explain the disorder.

**Methods:**

We detected the percentage of CD19+ B cells and serum immunoglobulin (IgG, IgA, and IgM) levels by flow cytometry and rate scatter immunoturbidimetry, and investigated the *BTK* mutation profile in 22 XLA patients using Sanger sequencing and real-time PCR .

**Results:**

We evaluated the clinical symptoms of 22 XLA patients and investigated genetic mutations present, identifying six novel mutations in the *BTK* gene: 2 missense mutations (c.23G > T and c.112 T > C), 2 frameshift mutations (c.522_523insC and c.1060delA), 1 large deletion (deletion of exon 2 to 5), and 1 splice-site mutation (c.1631 + 2 T > C). Prenatal diagnoses were performed in six families (F10, F11, F15, F18, F20 and F21), with the following results: the male fetus in Family 10 (F10) did not carry the c.922_923delGA mutation; the male fetus in Family 15 (F15) did not carry the c.1631 + 1G > T splicing mutation; the female fetus in Family 20 (F20) did not carry the c.1931 T > C mutation; the female fetus in Family 21 (F21) did not carry the large deletion mutation. Hence, these four fetuses are not likely to develop XLA. Male fetuses with c.1060delA and c.1684C > T mutations were identified in Family 11 and Family 18, respectively. The pregnant woman in F18 chose to terminate the pregnancy, whereas the pregnant woman in F11 chose to continue the pregnancy.

**Conclusion:**

We confirmed the diagnosis of 22 XLA patients from 22 unrelated families and detected six new pathogenic mutations. Prenatal diagnosis was performed in six families. Early genetic diagnosis and routine lifelong immunoglobulin replacement therapy can prevent and treat infections in XLA children, saving their lives.

## Background

X-linked agammaglobulinaemia (XLA, OMIM: 300755) is a rare immunodeficiency disease caused by defective B cell development and extremely low numbers of mature B cells [1]. The main clinical symptom of XLA is recurrent severe infection [2]. The estimated incidence of XLA is approximately 1:250,000, and the causative mutations are located in the Bruton’s tyrosine kinase (*BTK*) gene [3, 4]. The *BTK* gene is located at Xq21.3-Xq22; the gene is 37.5 kb and comprises 19 exons. The protein encoded by the gene is a cytoplasmic tyrosine kinase that contains five different functional domains: pleckstrin homology (PH), Tec homology (TH), Src homology 3 (SH3), SH2, and kinase (TK) domains [5]. The N-terminal PH domain binds to membrane phosphatidylinositol (3,4,5)-trisphosphate (PIP3), and the TH, SH3, and SH2 domains are involved in protein-protein interactions. Y223 and Y551 are two tyrosine phosphorylation sites in the SH3 and TK domains, respectively [6]. BTK activates many major signaling pathways, including the phosphoinositol-3 kinase (PI3K)-AKT pathway, phospholipase-C (PLC), protein kinase C, and nuclear factor kappa B (NF-kB) [7]. BTK also participates in B cell receptor (BCR) engagement by antigens and induces a range of protein interactions as well as recruitment of signaling molecules, resulting in B cell survival, proliferation and differentiation and the production of antibodies [8].

## Methods

### Patients and study design

From 2016 to 2019, 22 male XLA patients from 22 unrelated families in Henan Province of China were enrolled in this study. XLA was diagnosed according to the diagnostic criteria for XLA developed by the Joint European Society for Immunodeficiencies Committee [9]. After determining *BTK* gene mutations in the proband, the fetal villi or amniotic fluid of high-risk pregnant women were used for prenatal diagnosis. Mutation analysis of the fetal genome was carried out by DNA sequencing. The study was approved by the Ethics Committee of the First Affiliated Hospital of Zhengzhou University. The patients 16 years of age and over signed informed consent forms. A written informed consent was obtained from the parents or legal guardians of any participant under the age of 16.

### Routine immunological analysis

Serum was separated from 3 mL of peripheral venous blood without anticoagulant treatment. Immunoglobulins were examined by rate scatter immunoturbidimetry using a Siemens BN II automatic protein analyzer. CD19+ was detected with a FACSCanto II flow cytometer using 3 mL of EDTA-treated blood.

### Genetic testing

Genomic DNA was extracted from 2 mL of EDTA-treated peripheral venous blood from each proband and mother using Blood DNA Midi Kit D3494 (Omega Biotek, USA) with nucleic acid automatic extraction equipment (Eppendorf epMotion 5075 m, Germany). Amniotic fluid cell DNA was extracted and cleaned using QIAamp Blood DNA Midi Kit (250, Germany) and Genomic DNA Clean & Concentrator (Zymo Research, USA). The DNA sequence of the *BTK* gene obtained from the NCBI database was used as a reference. PCR amplification was carried out using relevant primers (Table S1) under conventional PCR conditions. The PCR product was confirmed by 2% agarose gel and was purified for two-way sequencing. The sequencing product was separated using an ABl3130xl gene sequencing machine. The sequencing results were compared by Chromas software to identify mutation loci. For novel mutations, Genome Aggregation Database (gnomAD) and the UCSC database were used. In addition, the large the *BTK* gene deletion was assessed using QuantStudio 5 Real-Time PCR System (ABI, USA).

## Results

### Clinical characteristics

Twenty-two families were enrolled in this study. The mean age of onset of XLA was 3 years, and the mean age of diagnosis was 7 years. The clinical infections present at the time of diagnosis are shown in Table 1. Of the types of infections, respiratory infection was the most common (*n* = 19, 78.9%), followed by sinusitis, sepsis, otitis media and central nervous system infection.

**Table 1:** Characteristics of 22 XLA male patients

Patients	Age at onset, y	Age at diagnosis, y	CD19+ B cells, %	IgG,g/l (5.66–14.25)	IgA,g/l(0.8–5)	IgM,g/l (0.3–2.09)	Clinical presentation
P1	1	2	1(6–25)	0.510	0.060	0.060	Pneumonia, herpetic stomatitis
P2	3	5	0(6–25)	< 0.810	< 0.330	0.300	Mycoplasmal pneumoniae
P3	6 months	4	0.00(5.0–18.0)	0.170	0.030	0.220	Sepsis, bilateral otitis media, sepsis
P4	14	15	1(6–25)	< 1.770	< 0.060	< 0.080	Infectious diarrhea, dystrophic anemia
P5	7 months	10 months	…	0.240	0.030	0.200	Bronchopneumonia, gastrointestinal dysfunction
P6	9	26	…	0.260	…	…	…
P7	2	7	0.23(6–25)	< 0.020	< 0.070	< 0.150	Central nervous system infection, epilepsy, upper respiratory infection, hydronephrosis
P8	3	9	0.0(5.0–18.0)	0.710	0.010	0.130	Bronchopneumonia, bronchiectasis, airway hyperresponsiveness, sinusitis
P9	3	13	0.00(5.0–18.0)	4.400	0.460	0.570	Bronchopneumonia, pleural effusion
P10	20 days	4	0.00(5.0–18.0)	5.640	0.110	0.050	Pneumonia, bronchiectasis
P11	5	5	0(6–25)	< 0.550	< 0.050	< 0.200	Acute upper respiratory tract infection, viral encephalitis, pneumonia
P12	7	8	1(6–25)	< 0.800	< 0.090	< 0.220	Bronchopneumonia
P13	3	3	0.00(5.0–18.0)	1.500	0.790	0.760	Bronchopneumonia, dilated cardiomyopathy, Vitamin k deficiency, Upper respiratory tract infection, sepsis
P14	2	7	0.04(5.0–18.0)	…	…	…	…
P15	2	3.5	…	0.100	0.000	0.040	Pneumonia
P16	1	10	0.00(5.0–18.0)	0.410	0.020	0.040	Recurrent cough, bronchiectasis, pulmonary infection, hepatitis B virus carrier
P17	9 months	2	0(6–25)	< 0.100	< 0.090	< 0.18	Pneumonia
P18	1	5	0.00(5.0–18.0)	0.100	0.020	0.040	Bronchopneumonia
P19	2	8	…	< 2.090	< 0.000	< 0.000	Pulmonary infection, bronchiectasis, sinusitis
P20	3	3	…	< 0.030	< 0.000	< 0.060	Pneumonia, iron-deficiency anemia, hypoalbuminemia
P21	6 months	8	…	0.300	…	…	…
P22	6 months	5	0.03(5.0–18.0)	< 0.740	< 0.000	< 0.130	Perianal abscess, anal fistula, chronic nasosinusitis, otitis media, sepsis

### Immunological features

As indicated in Table 1, all patients exhibited a very low percentage of CD19+ B cells and serum immunoglobulin (IgG, IgA, and IgM) levels at diagnosis. No patients got intravenous immunoglobulin (IVIG) substitution therapy before diagnosis. The percentage of CD19+ B cells in all patients was 0–1%, and ten of the sixteen patients had a B cell proportion of 0%. Nineteen of 21 patients had serum IgG concentrations of less than 2 g/L; two of 21 patients had serum IgG concentrations greater than 2 g/L but less than 5.66 g/L. The concentration of serum IgA (*n* = 19) in all patients was less than 0.8 g/L, and that of serum IgM in all patients was less than 0.3 g/L, except for patients 2, 9 and 13.

### *BTK* mutation analysis

**Fig. 1:**
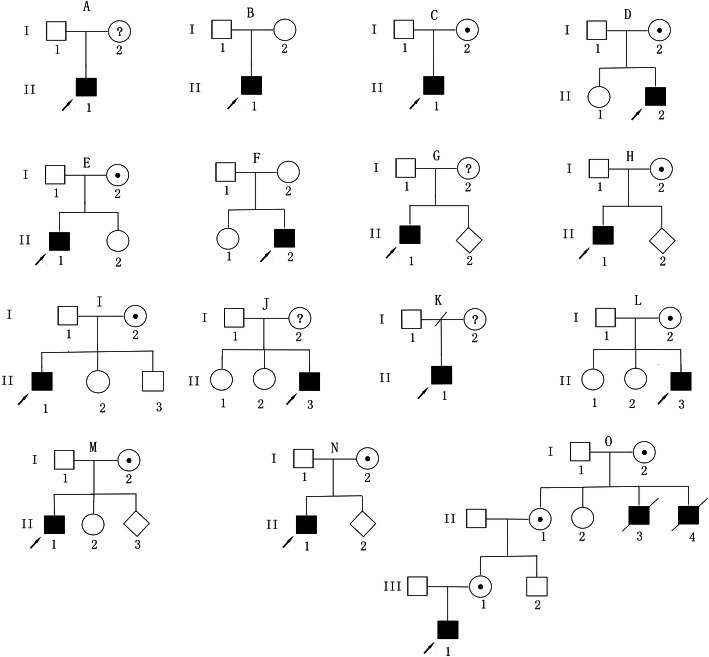
Heredity map of the family with the proband. (Each letter (A-O) represents a type of the pedigree. The details can be found in the right-most column of Table 2)

To confirm the diagnosis, mutation analysis of the *BTK* gene was performed (Table 2). Analysis of exons 1 to 19 and the flanking intronic regions revealed the presence of 22 different mutations in the 22 patients from 22 unrelated families, including 8 missense mutations, 4 nonsense mutations, 3 splice-site mutations, 5 frameshifts resulting in secondary premature termination, and 2 large deletions. Among them, 6 are novel mutations, including 2 missense mutations (c.23G > T and c.112 T > C), 2 frameshift mutations (c.522_523insC and c.1060delA), 1 large deletion (deletion of exon 2 to 5), and 1 splice-site mutation (c.1631 + 2 T > C). Genetic analysis of carrier status was conducted in 16 families with definitive *BTK* gene mutations, and 14 carriers with *BTK* gene mutations were identified. The mothers in the other six families were not tested because of death, divorce or subjective will. The pedigree of the above 22 families is shown in Fig. 1 and Table 2 (the right-most column).

**Table 2:** *BTK* gene mutations in 22 XLA patients from 22 unrelated families

Family	Patient	Localization	Domain	Nucleotide substitutions	Amino acid change	Type of mutation	Mother status	Pedigree ^**c**^
F1	P1	Exon 2	PH	c.23G > T ^**a**^	S8I	missense mutation	NE	A
F2	P2	Exon 2	PH	c.83G > A	R28H	missense mutation	NE	A
F3	P3	Exon 2	PH	c.112 T > C ^a^	S38P	missense mutation	NMD	B
F4	P4	Exon 2	PH	c.126 T > G	Y42*	nonsense mutation	carrier	C
F5	P5	Exon 6	TH	c.460 T > C	C154R	missense mutation	carrier	D
F6	P6	Intron 6	TH	c.520 + 5G > A	Splicing	Splicing	NE	…
F7	P7	Exon 7	Proline rich	c.522_523insC ^a^	P177Tfs*17	FS (stop)	carrier	E
F8	P8	Exon 8	SH3	c.763C > T	R255*	nonsense mutation	NMD	F
F9	P9	Exon 10	SH2	c.862C > T	R288W	missense mutation	carrier	D
F10	P10	Exon 11	SH2	c.922_923delGA ^b^	D308Lfs*14	FS (stop)	NE	G
F11	P11	Exon 12	SH2	c.1060delA ^a b^	T354Pfs*49	FS (stop)	carrier	H
F12	P12	Exon 13	SH2	c.1117C > A	L373I	missense mutation	carrier	I
F13	P13	Exon 14	Kinase	c.1184G > A	W395*	nonsense mutation	NE	J
F14	P14	Exon 15	Kinase	c.1439delG	G480Afs*4	FS (stop)	carrier	C
F15	P15	Intron 16	Kinase	c.1631 + 1G > T ^b^	Splicing	Splicing	carrier	H
F16	P16	Intron 16	Kinase	c.1631 + 2 T > C ^a^	Splicing	Splicing	NE	K
F17	P17	Exon 17	Kinase	c.1679delC	P560Qfs*10	FS (stop)	carrier	L
F18	P18	Exon 17	Kinase	c.1684C > T ^b^	R562W	missense mutation	carrier	M
F19	P19	Exon 18	Kinase	c.1901G > A	W634*	nonsense mutation	carrier	C
F20	P20	Exon 19	Kinase	c.1931 T > C ^b^	F644S	missense mutation	carrier	N
F21	P21	…	…	Deletion of exon 2 to 5 ^a b^	large deletion	large deletion	carrier	H
F22	P22	…	…	Deletion of exon 6 to 10	large deletion	large deletion	carrier	O

FS (stop): frameshift resulting in secondary premature termination

*NE* not examined, *NMD* no mutation detected

^a^: novel mutation

^b^:represent prenatal pedigree

^c^: Each letter (A-O) represents a type of the pedigree. All pedigrees of the families can be found in Fig. 1

*: represent the terminator

Four of 22 mutations identified in the *BTK* gene are located in the PH domain, 2 in the TH domain, 1 in the SH3 domain, 4 in the SH2 domain and 8 in the kinase domain. In addition, 2 mutations (the large exon deletion) are predicted to affect the translation of the protein.

### Prenatal diagnosis

**Fig. 2:**
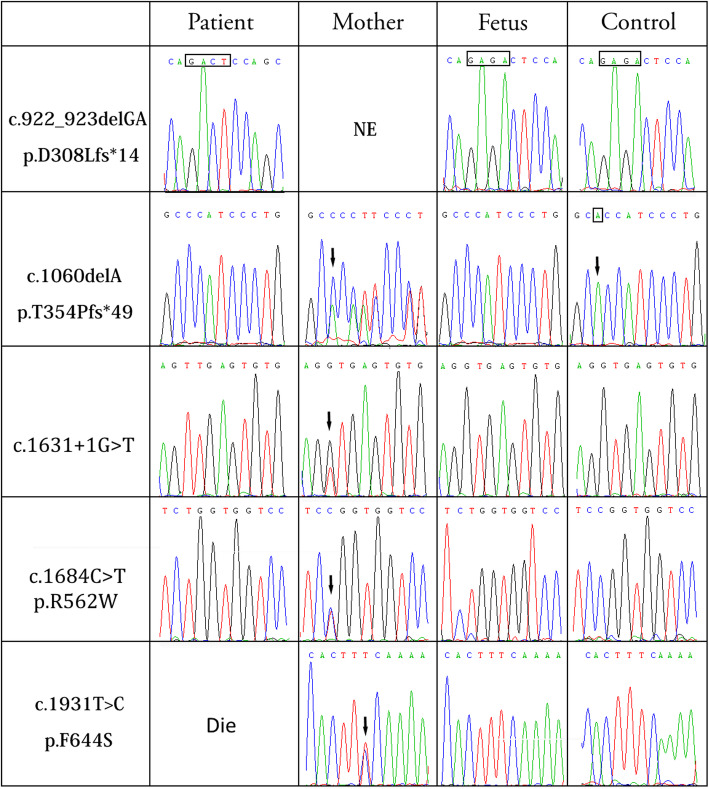
Sequence chromatograms of *BTK* variants in five prenatal pedigrees and controls

**Fig. 3:**
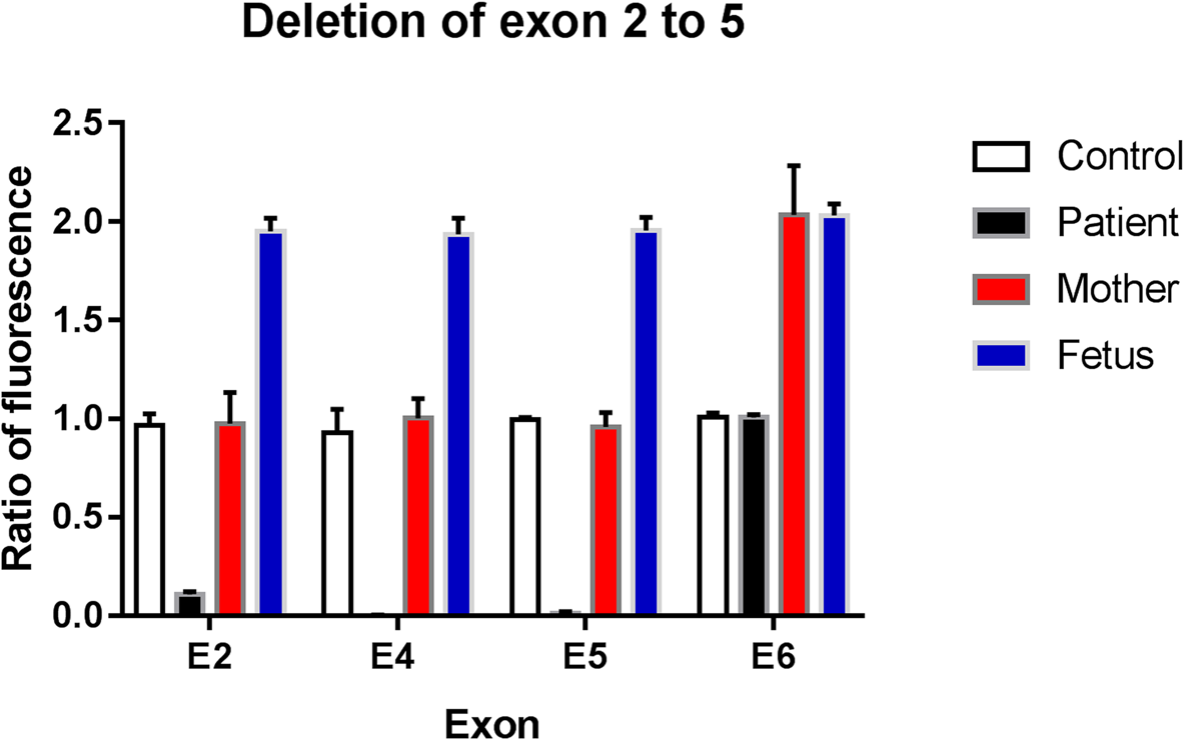
Real-time PCR results of *BTK* gene in Family 21 and the male control. (The deletion of exon 2, 4 and 5 was hemizygous in the affected proband, and heterozygous in the mother. The female fetus and the male control did not carry the large deletion mutation)

Six families (F10, F11, F15, F18, F20 and F21) who received prenatal diagnosis (Figs. 2 and 3); we confirmed that the fetal villi or amniotic fluid samples had not been contaminated by the material from the mother. The male fetus in Family 10 did not carry the p.D308Lfs*14 mutation, and the male fetus in Family 15 did not carry the c.1631 + 1G > T splicing mutation. In Family 20, the female fetus did not carry the c.1931 T > C mutation, and in Family 21, the female fetus did not carry the large deletion mutation (Figs. 2 and 3). None of these four fetuses are likely to develop XLA patients in the future. The above four families chose to continue the pregnancy after genetic counseling. Umbilical cord blood was collected for genetic diagnosis after full-term delivery, and the results were consistent with the prenatal diagnosis. According to telephone follow-up after 1 year, the general development of the infants was normal. Male fetuses in Family 11 and Family 18 carried p.T354Pfs*49 and p.R562W mutations, respectively. After genetic counseling, the pregnant woman in Family 18 chose to terminate the pregnancy, and DNA analysis of the tissues was consistent with the prenatal diagnosis results. Conversely, the pregnant woman in Family 11 chose to continue the pregnancy.

## Discussion

Here, we review the clinical data of 22 male XLA patients from 22 unrelated Chinese families in Henan Province. Our results showed that all patients had typical clinical presentations, including recurrent infections and hypogammaglobulinemia with a significant decrease or near complete absence of B cells in the peripheral blood. Respiratory infection was the main clinical feature of the patients in this study, consistent with previous findings [10]. The mean age at diagnosis was 7 years, which is higher than in a reported series from the United States [11] but similar to the series reported from other provinces in China [12]. There was also a considerable delay in the diagnosis of these patients, especially Patient 6, whose diagnosis of XLA was confirmed after 17 years, when we finally worked him up for this study. This may be due to the poor understanding of XLA among Chinese physicians.

The *BTK* gene is located at Xq21.3-Xq22 and encompasses 37.5 kb, which contains 19 exons. The first exon of this gene is a noncoding region, and the remaining 18 exons encode the BTK protein [13]. To date, 911 mutations in the *BTK* gene related to XLA have been deposited in the Human Gene Mutation Database (http://www.hgmd.cf.ac.uk/ac/gene.php?gene=BTK). The spectrum of these mutations includes missense mutations, nonsense mutations, splice site mutations, insertions, and deletions, with missense mutation being the most common. It has already been shown that these mutations can occur in the exons, introns, and promoters of the *BTK* gene [14, 15]. In this study, six novel mutations were found (Table 2): three are point mutations, one is an insertion, and two are deletions. Other mutations that have previously been reported are recurrent mutations.

Patient 7 and Patient 20 died of serious infection during the study follow-up. Patient 6 and Patient 15 were lean, with BMIs of 18.6 kg/m^2^ (1.78 m, 59 kg) and 13.8 kg/m^2^ (1.29 m, 23 kg), respectively. Patient 21 and Patient 22 experienced multiple repeated infections from the time they were 6 months old. Their mutations are large deletions resulting in loss of fragments of the peptide chain, with consequent loss of BTK protein function. The serious condition of these two patients was greatly alleviated once their diagnosis was established and IVIG treatment was initiated. This confirms, as previously reported, the correlation between the patient’s genotype and phenotype in XLA [16]. For Patient 5, the age of onset and diagnosis of XLA was at five years of age. According to his mother, the patient had no signs of a weakened immune system before five years. He has received immunoglobulin injections twice a year and is in reasonable health. The second male fetus harbors the BTK p.T354Pfs*49 mutation, which is the same mutation carried by his brother (Family 11). Considering the phenotype of the proband, this family chose to continue the pregnancy. Therefore, variable XLA disease manifestation may result depending on the types and sites of *BTK* gene mutations.

XLA is an X-linked recessive genetic disease in which cases are usually male and carriers female. In general, it is very important to screen suspected carriers or cases in the families of patients. The young aunt of Patient 16 carried the c.1631 + 2 T > C mutation and the sister of Patient 17 p.P560Qfs*10. Carriers can undergo PGD (preimplantation genetic diagnosis) to know in advance their possibility of conceiving a child with XLA. As a result, some carriers may decide not to have a child. We found that the mothers of the probands in Family 3 and Family 8 did not carry the mutation; hence, p.S38P and p.R255* are likely de novo mutations.

## Conclusion

In summary, we confirmed the diagnosis of 22 XLA patients from 22 unrelated families by testing for *BTK* gene mutations and discovered six new XLA mutations. We also performed prenatal diagnosis of XLA in six susceptible families. Early genetic diagnosis and routine lifelong immunoglobulin replacement therapy can prevent and treat infections in XLA children and save their lives.

## Supplementary information

**Additional file 1: Table S1.***BTK* gene primers.

## Data Availability

The datasets generated during the current study are not publicly available because it is possible that individual privacy could be compromised. Ensembl database (http://grch37.ensembl.org/Homo_sapiens/Transcript/Exons?db=core;g=ENSG00000010671;r=X:100604438-100641183;t=ENST00000308731) and NCBI database (https://www.ncbi.nlm.nih.gov/nuccore/NM_000061) were used in our study.

## Abbreviations

XLA: X-linked agammaglobulinemia
BTK: Bruton’s tyrosine kinase
PH: Pleckstrin homology
TH: Tec homology
SH: Src homology
PIP3: Phosphatidylinositol (3,4,5)-trisphosphate
BCR: B cell receptor
PLC: Phospholipase-C
NF-kB: Nuclear factor kappa B
PI3K: Phosphoinositol-3 kinase
gnomAD: Genome Aggregation Database
IgA: Immunoglobulin A
IVIG: Intravenous immunoglobulin
BMI: Body Mass Index
PGD: Preimplantation genetic diagnosis
