# Supplementary material for: Clinical characteristics and prenatal diagnosis for 22 families in Henan Province of China with X-linked agammaglobulinemia (XLA) related to Bruton’s tyrosine kinase (BTK) gene mutations
Source: BMC Med Genet. 2020 Jun 17;21:131. doi: 10.1186/s12881-020-01063-5 (PMC7302398; doi:10.1186/s12881-020-01063-5)
Supplement: Supplementary file 1 — Additional file 1: Table S1.BTK gene primers. [file 12881_2020_1063_MOESM1_ESM.docx]

Table S1: *BTK* gene primers.

| **Name** | **Forward Primer** | **Reverse Primer** |
| --- | --- | --- |
| *BTK*-E1 | AAGGGAACTGAGTGGCTGTGAAAG | AGGGGTCCCAAGCAAGGTACAT |
| *BTK*-E2 | GGGGGAACCAAGAGGGATGAG | TCCCCTCCTCCTACCAACGAAAATT |
| *BTK*-E3 | CCCCCACATGACAGGTCCT | CAGCATCACCAGTCTATTTACAG |
| *BTK*-E4 | GAAAAGAGCAATGCATCAACCAATA | CCCCACCACCCCTTCTAATTGT |
| *BTK*-E5 | TCTCCTTTTTTCTGAATCCTAACT | TCCTCTTCCTTCCTTTCCTTCTTTC |
| *BTK*-E6 | GGGGGCAGTTGCTTGAAGTTC | CCCCCAGGACCCTTTGTTTAG |
| *BTK*-E7 | TTCCTAAAGTGCATTCCATATC | TGCCAAGTCCCAGGGTAATTCTAAG |
| *BTK*-E8 | TGCCTGATTTGGGTGTCTTGAGTAA | GCTGCACGCTGGGAAGTAC |
| *BTK*-E9 | ACTGGCTCACTGCCTAACCA | CATAAATGGAGGGTGTTTGGGG |
| *BTK*-E10 | TGCCCTAGTCCCTGATCTCTTC | GGCCCTCAGTTCAAGATCCTCAC |
| *BTK*-E11E12 | GCCCGGCCTGCACCTTTTAATA | CCTGCATTGCTTATCCTGGTGTC |
| *BTK*-E13 | GGGCTGGGAGGTGGGAATATAT | AGCCCTTTATGAATCTATGTTTT |
| *BTK*-E14 | TCCCGTTTCTGAGATTGACTTAAG | CCCCCAAATGCTACTGAGATG |
| *BTK*-E15 | GCCCCTTTATTGCTATTATGC | GCCCCCCTCAACCATGTATG |
| *BTK*-E16 | ACGGAGTCTCACTGGTCTCT | GAAAGATCGGCAGAAAACGCT |
| *BTK*-E17 | GCCAAGAAAACGTAAGCAAATATC | GGCAGAGATCATGTCCAGTTTAGTT |
| *BTK*-E18 | GCGGGGAACCAACTGATTCTA | TGTGTGCAGCTATCAGTCTTTG |
| *BTK*-E19 | AGCCATTTTTCACTTGAGGATCCA | GGGGCCTTTTTGTATTGAGTG |
